# Safety of neoadjuvant radiotherapy or chemoradiotherapy combined with immunotherapy across solid tumors: Systematic review and meta-analysis protocol

**DOI:** 10.1371/journal.pone.0357205

**Published:** 2026-08-31

**Authors:** Irtaza Tahir, Susheen Mahmood, Minoo Aminnejad, Elena Parvez

**Affiliations:** 1 Department of Surgery, Faculty of Health Sciences, McMaster University, Hamilton, Ontario, Canada; 2 Michael G. DeGroote School of Medicine, McMaster University, Hamilton, Ontario, Canada; All India Institute of Medical Sciences, INDIA

## Abstract

**Objective:**

The aim of this systematic review and meta-analysis is to evaluate whether neoadjuvant radiotherapy or chemoradiotherapy, combined with immune checkpoint inhibitors for adults with resectable solid tumors, increases the risk of severe treatment-related toxicity and organ-specific adverse events compared with radiotherapy or chemoradiotherapy alone.

**Methods:**

A systematic review and meta-analysis will be performed and reported in accordance with the Preferred Reporting Items for Systematic Reviews and Meta-Analyses. We will identify eligible studies published in MEDLINE, Embase, and the Cochrane Central Register of Controlled Trials from January 2015 through March 2026. Clinical trial registries and major oncology conference proceedings will be searched, and references of included studies and relevant prior systematic reviews will be reviewed. Eligible studies will include randomized and observational cohorts of adults with resectable solid tumors receiving neoadjuvant radiotherapy or chemoradiotherapy combined with immune checkpoint inhibitors and reporting at least one prespecified safety outcome. Outcomes of interest include grade ≥ 3 treatment-related adverse events, grade ≥ 3 immune-related adverse events (overall and by organ system), treatment discontinuation, treatment-related death, requirement for systemic immunosuppression, radiotherapy site toxicities, and 90-day perioperative complications after surgery. Where appropriate, comparative data will be pooled using random-effects models and explored in subgroup analyses by tumor site, fractionation approach, and timing of immune checkpoint inhibitor delivery relative to radiotherapy. The protocol has been registered on the International Prospective Register for Systematic Reviews (PROSPERO CRD420261322883).

**Discussion:**

Neoadjuvant radiotherapy and chemoradiotherapy are established components of curative-intent care across multiple solid tumors, and immune checkpoint inhibitors are increasingly used perioperatively. However, safety data for combined neoadjuvant radio-immunotherapy are fragmented across tumor sites and treatment schedules, limiting clear estimates of severe and organ-specific toxicity. This study will synthesize the available evidence on toxicity and perioperative complications and clarify safety risks, identify settings where toxicity may be concentrated, and highlight evidence gaps that should be addressed in future prospective comparative trials.

## Introduction

Radiotherapy (RT) is traditionally viewed as a local cytotoxic modality, but it also exerts significant immunomodulatory effects within the tumor microenvironment. By inducing cell death, RT releases tumor-associated antigens and damage-associated molecular patterns, which are taken up by dendritic cells and other antigen-presenting cells (APCs), initiating adaptive immune responses [1]. RT also promotes local inflammation by increasing type I interferons, chemokine release, and vascular permeability, thereby facilitating T-cell infiltration into tumors [1].

When combined with immune checkpoint inhibitors (ICIs), these effects may be amplified. ICIs such as anti–PD-1, anti–PD-L1, and anti–CTLA-4 antibodies release inhibitory brakes on T cells, allowing RT-induced priming to translate into durable systemic immunity [1]. This synergy has been linked to the abscopal effect, wherein regression of non-irradiated lesions is observed following local RT in the presence of systemic immune activation [2]. Chemotherapy, when included as part of neoadjuvant chemoradiotherapy (nCRT), further contributes by enhancing immunogenic cell death and reducing immunosuppressive populations, potentially deepening synergy with ICIs [3].

The neoadjuvant setting provides a compelling context for radio-immunotherapy. Treating the intact primary tumor allows maximal antigen release, and immune priming before surgical resection may help eradicate micrometastatic disease. Moreover, radiotherapy while the primary is in situ can help minimize radiation-induced toxicity to surrounding non-malignant tissues. Early studies across tumor types including rectal cancer, esophageal carcinoma, and soft-tissue sarcoma suggest that adding ICIs to standard nCRT is feasible and may enhance pathologic responses without prohibitive toxicity [4–7]. A recent single-arm systematic review in dMMR/MSI-H rectal cancer reported encouraging pCR rates with PD-1/PD-L1 blockade plus nCRT, while highlighting that grade ≥3 toxicity rates remained in the range of ~20% [4]. In sarcoma, the randomized SARC032 trial confirmed that perioperative pembrolizumab with preoperative RT was safe and associated with improved disease-free survival compared with RT alone [5]. Similarly, esophageal trials (PERFECT and PALACE) demonstrated acceptable safety profiles when ICIs were combined with CROSS-like regimens [6,7].

Despite these promising signals, a systematic synthesis of safety outcomes across cancer types is lacking. Moreover, toxicity reporting is heterogeneous, with variation in Common Terminology Criteria for Adverse Events (CTCAE) versions (with v5 published 2017 and v6 published 2025) and variable attribution of adverse events to immunotherapy (i.e., immune-related adverse events (irAEs)) or RT. Importantly, organ-specific toxicities such as pneumonitis after thoracic RT or dermatitis in large-field RT are of concern when ICIs are combined with RT [8]. Therefore, a focused review is needed to clarify the risks of neoadjuvant RT (nRT)/nCRT in combination with immunotherapy.

### Rationale

A systematic review to synthesize safety evidence will clarify whether nRT/nCRT combined with ICIs increases severe or organ-specific toxicities compared with nRT/nCRT alone, and whether risks differ by timing (concurrent vs sequential), fractionation, or tumor site.

### Research question

Among adults with resectable solid tumors, does nRT or nCRT combined with ICIs increase the risk of adverse events compared with nRT or nCRT alone?

### Hypothesis

1. nRT/nCRT in combination with ICI increases specific organ-related AEs (e.g., pneumonitis in thoracic fields) but not overall grade ≥3 AE rates compared with nRT/nCRT alone
2. Safety outcomes will differ by tumor site, nRT fractionation, and timing of ICI administration.

## Materials and methods

We will perform a systematic review and meta-analysis comparing the impact of nRT or nCRT combined with ICI vs nRT/nCRT alone on safety. We will report our results according to Preferred Reporting Items for Systematic Reviews and Meta-Analyses (PRISMA) guidelines [9]. Below we outline our methodology for this review which has been reported according to PRISMA-P [10].

### PICO

- Population – Adults (≥18 y) with resectable solid tumors undergoing neoadjuvant therapy.
- Intervention – nRT or nCRT combined with ICIs (PD-1, PD-L1, CTLA-4, or LAG-3).
- Comparator – nRT or nCRT alone
- Outcomes –
  - Primary: Grade ≥3 treatment-related adverse events (trAEs) per CTCAE v3–v6.
  - Secondary:
    - Grade ≥3 immune-related AEs (irAEs) overall and by organ (pneumonitis, colitis, hepatitis, endocrinopathy, myocarditis, etc.)
    - Any-grade trAEs/irAEs
    - Treatment-related death
    - Discontinuations due to AEs
    - High-dose steroids or other immunosuppression for irAEs
    - RT-related site toxicities (e.g., esophagitis, proctitis, cystitis, dermatitis)
    - Perioperative complications (Clavien-Dindo ≥III, wound issues, anastomotic leaks, 30/90-day mortality)

### Intervention

The intervention of interest is neoadjuvant radiotherapy or chemoradiotherapy administered in combination with an immune checkpoint inhibitor

- Radiotherapy will include any external-beam technique (e.g., 3D-CRT, IMRT/VMAT, proton therapy, stereotactic body radiotherapy [SBRT], stereotactic radiosurgery [SRS]) delivered to the intact primary tumor and/or regional nodes in the preoperative setting. Intraoperative brachytherapy alone or radiopharmaceuticals will be excluded.
- Chemoradiotherapy refers to RT delivered concurrently with systemic cytotoxic therapy according to disease-specific standards (e.g., long-course nCRT in rectal cancer, CROSS regimen in esophageal cancer)
- Immunotherapy will include ICIs targeting PD-1, PD-L1, CTLA-4, or LAG-3, administered either as monotherapy or in combination. Dual-checkpoint blockade will be eligible.
- Timing of RT–ICI delivery may be concurrent (calendar overlap of RT and ICI) or with delivery of ICI in an induction or consolidation manner, prior to surgical resection
- Fractionation approaches of interest will include: conventional fractionation, hypofractionation, short-course RT, and SBRT/SRS

### Control

Eligible comparator arms will include

1. nRT or nCRT without immunotherapy, representing the current standard of care in several solid tumors

Comparator arms must involve a preoperative component with curative-intent surgery planned. Trials using adjuvant-only radiotherapy or ablative non-surgical RT without a clear surgical intent will be excluded. Where multi-arm or platform trials are reported, pairwise comparisons will be extracted separately.

### Outcome definitions

#### Primary outcome.

- Grade ≥3 treatment-related adverse events (trAEs): Incidence of severe or life-threatening AEs occurring during the neoadjuvant period through 90 days postoperatively, graded according to the CTCAE. Grade ≥3 trAEs will be treated as a binary outcome (event vs no event) within each study (number of participants with ≥3 trAE/ total participants).

#### Secondary outcomes.

- Immune-related adverse events (irAEs): Grade ≥3 and any-grade events attributed to checkpoint inhibitor use, including but not limited to pneumonitis, colitis, hepatitis, endocrinopathies, dermatologic toxicities, myocarditis, nephritis, and neurologic events.
- Any-grade trAEs: Incidence of all-grade treatment-related AEs.
- Treatment-related death (Grade 5 AEs): Fatal AEs judged related to study treatment.
- Treatment discontinuation due to toxicity.
- Requirement for systemic steroids or immunosuppressive agents for AE management.
- Radiation site–specific toxicities: Including radiation dermatitis, esophagitis, proctitis, cystitis, pneumonitis, etc.
- Perioperative complications: Postoperative complications reported within 30 or 90 days, preferentially graded using the Clavien–Dindo classification. Specific complications of interest include wound complications, anastomotic leak, cardiopulmonary events.
- 30-day and 90-day postoperative mortality,

### Information sources

The following databases, will be searched:

1. Medline
2. Embase
3. Cochrane Central Register of Controlled Trials (CENTRAL)

The search will be restricted to January 2015 to March 31, 2026, as immune checkpoint inhibitors first entered clinical oncology practice during the 2010–2015 period, and large studies of neoadjuvant radio-immunotherapy combinations in non metastatic settings were not reported prior to this. No language restrictions will be imposed during the search. The search strategy for Medline is included in Supplement 1.

All searches will be saved using an account established in each search database. Databases such ClinicalTrials.gov will be searched for ongoing studies. Abstracts from major oncology conference proceedings (ASCO, ASTRO, ESMO, AACR) will be searched given the relative novelty of combination neoadjuvant radiotherapy and immunotherapy. Other sources of grey literature will be limited to clinical trial results reported in theses, dissertations, and conference papers. The references of studies meeting inclusion criteria as well as previous pertinent systematic reviews will be searched manually to include all relevant articles.

### Study selection

Two reviewers will independently evaluate the systematically searched titles and abstracts using a web based screening software (Covidence). Discrepancies that occur at the title and abstract screening phases will be resolved by inclusion of the study. At the full-text screening stage, discrepancies will be resolved by consensus between the reviewers. If disagreement persists, an additional reviewer will be consulted.

### Eligibility criteria

#### Inclusion criteria.

- Randomized trials, controlled trials, prospective cohort or retrospective cohort,
- Studies evaluating adult patients 18 years of age or older
- Solid tumors receiving neoadjuvant RT/nCRT + ICI
- Must report ≥1 prespecified safety outcome

All four criteria must be satisfied for a study to be included.

#### Exclusion criteria.

- Wrong population – e.g., metastatic unresectable cancer, locally advanced unresectable cancer, treatments delivered in an adjuvant setting only, primary definitive therapy regimen, intraoperative radiotherapy, brachytherapy, radiopharmaceuticals, pediatric population
- Wrong study type – i.e., Systematic reviews, meta-analysis, case series, case study, surveys, letters, editorials, or any other type of study not reporting primary data
- Wrong outcome – i.e., neither primary nor secondary outcomes are a safety measure

### Data management and items

Two reviewers will independently conduct data extraction into a data collection form designed *a priori*. The form will first be piloted, then extraction will be done independently in duplicate. Discrepancies will be reviewed in detail and discussed until consensus is reached and if necessary adjudicated by a third reviewer.

The extracted data will include:

1. Study characteristics: author, year of publication, country, study period, study design, number of centers, study inclusion criteria, study exclusion criteria, sample size, description of intervention, description of comparator, reported outcomes, statistical methods, duration of follow up
2. Patient demographics: age, sex, performance status (ECOG or Karnofsky), body mass index (BMI), smoking status, comorbidities, autoimmune disease history, prior cancer therapy, baseline immunosuppression (e.g., corticosteroids), and ASA score
3. Disease characteristics: tumour site (e.g., rectal, esophageal, sarcoma, head and neck, lung, etc), histologic subtype, TNM staging, resectability criteria, and biomarker status (e.g., MMR/MSI status, PD-L1, expression, TMB) if available
4. Intervention and Comparator details:
  a. **Immunotherapy:** agent(s), class (PD-1, PD-L1, CTLA-4, LAG-3), dose, route, cycle length, number of cycles delivered preoperatively, and whether given as monotherapy or in combination
  b. **Radiotherapy:** total dose, number of fractions, technique (3D-CRT, IMRT/VMAT, proton therapy, SBRT, SRS), field location (thoracic vs non-thoracic), and calculated BED_10 if available
  c. **Chemotherapy (if part of nCRT):** regimen (e.g., 5-FU, capecitabine, FOLFOX, CAPOX, cisplatin-based), timing relative to nRT and ICI (induction, concurrent, consolidation)
  d. **Sequencing:** concurrent versus sequential (with exact interval in days if reported)
  e. **Comparator arms:** nRT/nCRT alone or other neoadjuvant standard; extracting the same details as above
  f. **Surgical details:** Type of surgery (e.g., low anterior resection, esophagectomy, sarcoma resection, etc.), approach (open, laparoscopic, robotic), interval from last neoadjuvant treatment to surgery, diversion or stoma formation, anastomotic technique, and intraoperative complications
  g. **Adjuvant treatment:** Postoperative systemic therapy, adjuvant ICI, or adjuvant RT, when applicable
5. Safety Outcomes
  a. **Adverse events (AEs):** Any-grade and grade ≥3 trAEs and irAEs, graded according to CTCAE v3–v6, harmonized to v5 definitions.
    i. For synthesis, events will be harmonized to CTCAE v5 definitions, which are currently most widely adopted in oncology trials. Harmonization will follow the approach described by Trotti et al. and subsequent comparative analyses of CTCAE versions, where terminology differences are reconciled by mapping older descriptors onto v5 categories (e.g., “Grade 3 dyspnea limiting self-care” in v3 maps directly to Grade 3 pneumonitis in v5) [11]
  b. **Specific toxicities:** pneumonitis, colitis/diarrhea, hepatitis, endocrinopathies, dermatologic reactions, nephritis, myocarditis, neurologic events, esophagitis, proctitis, cystitis, radiation dermatitis, etc
  c. **Treatment-related death (grade 5 AEs)**
  d. **Treatment discontinuation due to AEs**
  e. **Steroid or immunosuppressive therapy use** for AE management
  f. **Perioperative complications:** graded by Clavien–Dindo when available, [12] including wound complications, anastomotic leak, cardiopulmonary events, readmission, length of stay, and 30-/90-day postoperative mortality

### Data synthesis and statistical considerations

All eligible studies will be included in a structured narrative synthesis, organized by cancer type, intervention, comparator, and reported safety outcomes. A quantitative synthesis will be undertaken when at least three studies report comparable outcomes. For dichotomous safety outcomes such as grade ≥3 treatment-related adverse events, pneumonitis, or treatment-related death, pooled effect estimates will be generated using an inverse variance random-effects. In instances where event rates are rare, a random-effects model with continuity correction will be applied. Results will be expressed as risk ratios (RRs) with corresponding 95% confidence intervals (CIs). When available, adjusted effect estimates (e.g., RR from multivariable models) will be extracted and synthesized narratively, while unadjusted pooled estimates will be presented separately. Continuous outcomes, such as time to onset of adverse events, will be summarized as mean differences (MDs) with 95% CIs; when studies report medians and interquartile ranges, means and standard deviations will be estimated using validated methods (Wan et al.) [13]. Missing SD data will be calculated according to the prognostic method [14]. Assessment of heterogeneity will be completed using the inconsistency (I^2^) statistic. An I^2^ greater than 40% will be considered to represent at least moderate heterogeneity. Potential publication bias will be explored using funnel plots where at least 10 studies contribute to an outcome in the meta analysis. Statistical significance will be defined a priori as a two-sided *p* value <0.05. Statistical analysis will be performed using R software.

### Planned subgroup analyses

If sufficient data are available, subgroup analyses will be performed to explore potential sources of heterogeneity:

1. Timing of ICI–nRT delivery: concurrent, induction, or consolidation.
  a. The sequencing of immunotherapy and radiotherapy is a prespecified subgroup because treatment timing may significantly modify both toxicity risk and biological interaction. Induction ICI (ICI before nRT) may prime systemic T-cell activation before radiation induced antigen release. When ICI are delivered concurrently with RT, patients experience overlapping inflammatory pathways triggered by radiation-induced antigen release and immune checkpoint blockade, which may potentiate immune activation but also increase the incidence and severity of acute toxicities such as pneumonitis, dermatitis, or colitis. Consolidation ICI (ICI after nRT) may reduce overlapping acute toxicity during nRT while still leveraging post-nRT antigen presentation, although delayed or subacute immune toxicities may occur closer to surgery depending on interval and dosing. Distinguishing induction, concurrent, and consolidation strategies is clinically important because each represents a different balance between potential immunologic synergy, tolerability, and readiness for timely surgery. Clarifying whether adverse event rates differ by timing will inform regimen design, eligibility criteria, and perioperative monitoring in future trials.
2. Tumor site: rectal vs esophageal vs sarcoma vs other solid tumors.
  a. As safety outcomes are often organ- and site-specific, subgroup analysis by tumor site will be conducted. Pooling data across heterogeneous cancer types without stratification may obscure clinically meaningful differences in toxicity profiles. Stratifying by tumor site allows adverse events to be interpreted within the context of underlying anatomical vulnerability, radiation fields, and treatment paradigms. This analysis will help determine whether immunotherapy-associated toxicities represent a generalized effect of combined modality therapy or are concentrated within specific disease settings, thereby improving the clinical interpretability of pooled safety estimates.
3. Radiotherapy backbone: Radiotherapy vs Chemoradiotherapy.
  a. The addition of concurrent cytotoxic chemotherapy may modify both toxicity risk and the interaction with immune checkpoint inhibition. Chemotherapy can increase baseline rates of grade ≥3 adverse events through myelosuppression, mucositis, gastrointestinal toxicity, and perioperative complications, and it may also contribute to immune-mediated toxicity risk by amplifying systemic inflammation or impairing tissue repair. In contrast, radiotherapy-only regimens may have a narrower toxicity profile that is more field and dose-dependent, potentially altering the frequency and pattern of events. Because RT and nCRT are used in different disease settings and delivered with different schedules, pooling them without stratification may obscure clinically important differences in safety outcomes attributable to the treatment backbone rather than checkpoint blockade. Separating RT from nCRT will improve interpretability of pooled estimates and help determine whether observed severe or organ-specific toxicities are driven primarily by the addition of chemotherapy, the radiotherapy, or the combined modality interaction with immunotherapy.

Additional exploratory subgroups may be considered depending on data availability, such as by immune checkpoint inhibitor class.

### Planned sensitivity analyses

Sensitivity analysis will include:

1. Excluding studies judged to be at high risk of bias (RoB 2.0 for RCTs, ROBINS-I for observational studies).
2. Restricting analyses to studies that graded adverse events using CTCAE v5 or higher.
3. Restricting analysis by when the adverse event was captured, restricting studies that report events up to surgery only, to assess whether perioperative complications alter safety estimates.
4. Restricting analyses by study design, repeating meta-analyses including RCTs only and observational studies only to assess whether pooled safety estimates differ by design and to evaluate the robustness of findings to potential confounding in non-randomized data

### Risk of bias assessment

Risk of bias for RCTs will be assessed using the Cochrane Risk of Bias Tool for RCTs 2.0 [15]. Risk of bias for observational studies will be assessed using the Risk of Bias in Non-randomized Studies of Interventions (ROBINS-I) assessment tool [16]. Two reviewers will assess the risk of bias independently. Discrepancies will be discussed amongst the reviewers until consensus is reached. Risk of bias figures will be created with RoBvis [17].

### Certainty of evidence

Certainty of evidence for each critical and important outcome will be assessed using the Grading of Recommendations Assessment, Development and Evaluation (GRADE) approach [18]. Two reviewers will assess the certainty of evidence and discrepancies will be discussed amongst the reviewers until consensus is reached. The certainty of the evidence will be rated as high, moderate, low, or very low for each outcome across the body of evidence after consideration of the GRADE domains of risk of bias, inconsistency of results, indirectness of evidence, imprecision, and publication bias. Where randomized and non-randomized evidence contribute to the same outcome, these bodies of evidence will be considered separately and integrated where appropriate in accordance with GRADE guidance. Certainty assessments will be undertaken for all key review outcomes, including grade ≥3 treatment-related adverse events, grade ≥3 immune-related adverse events, treatment discontinuation due to toxicity, treatment-related death, requirement for systemic immunosuppression, radiotherapy site-specific toxicities, and perioperative complications, etc. The rationale for each certainty judgment, including reasons for any downgrading and, where applicable, upgrading, will be documented transparently. Results will be presented in a GRADE evidence profile and Summary of Findings table generated using GRADEpro GDT software [19].

Study screening and data extraction are ongoing, data collection is expected to be completed by July 2026, and results are expected by September 2026.

## Discussion

Neoadjuvant radiotherapy and chemoradiotherapy remain standard components of curative-intent management across multiple solid tumors, and immune checkpoint inhibitors are increasingly being integrated into perioperative treatment strategies. While early-phase trials and disease-specific studies suggest that combining nRT/nCRT with ICIs is feasible, safety data remain fragmented across tumor sites and reported with substantial heterogeneity in adverse event definitions, attribution, and CTCAE versioning. The aim of this systematic review and meta-analysis is to synthesize the available evidence on treatment-related and immune-related toxicities associated with neoadjuvant nHRT/nCRT plus ICIs compared with appropriate control regimens. By consolidating organ-specific and severe adverse event outcomes, this review will help clarify whether toxicity risks differ by tumor site, fractionation, and timing of ICI delivery, and will support clinicians in balancing perioperative safety with potential oncologic benefit. Ultimately, these findings will identify knowledge gaps, inform trial design and eligibility criteria, and guide future prospective studies needed to establish safe, generalizable neoadjuvant radio-immunotherapy approaches.

## Supporting information

S1 File Search strategy from Ovid MEDLINE. (DOCX)

S2 File PRISMA-P Checklist. (DOCX)
