# Supplementary material for: Safety of neoadjuvant radiotherapy or chemoradiotherapy combined with immunotherapy across solid tumors: Systematic review and meta-analysis protocol
Source: PLoS One. 2026 Aug 31;21(8):e0357205. doi: 10.1371/journal.pone.0357205 (PMC13529006; doi:10.1371/journal.pone.0357205)
Supplement: Supplementary file 1 [file pone.0357205.s001.docx]

**S2 File.** Search strategy from Ovid MEDLINE

1	exp Radiotherapy/

2	exp Radiotherapy, Intensity-Modulated/

3	exp Proton Therapy/

4	exp Radiosurgery/

5	radiotherap*.ti,ab,kw.

6	radiation therap*.ti,ab,kw.

7	irradiat*.ti,ab,kw.

8	intensity-modulated radiotherap*.ti,ab,kw.

9	IMRT.ti,ab,kw.

10	volumetric-modulated arc therap*.ti,ab,kw.

11	VMAT.ti,ab,kw.

12	three-dimensional conformal radiotherap*.ti,ab,kw.

13	3D conformal radiotherap*.ti,ab,kw.

14	3D-CRT.ti,ab,kw.

15	3DCRT.ti,ab,kw.

16	stereotactic body radiotherap*.ti,ab,kw.

17	SBRT.ti,ab,kw.

18	stereotactic radiosurg*.ti,ab,kw.

19	(proton* adj2 (therap* or radiotherap*)).ti,ab,kw.

20	chemoradiotherap*.ti,ab,kw.

21	chemo-radiotherap*.ti,ab,kw.

22	chemo-radiation.ti,ab,kw.

23	chemoradiation.ti,ab,kw.

24	CRT.ti,ab,kw.

25	exp Immune Checkpoint Inhibitors/

26	exp Immunotherapy/

27	(("checkpoint" or "immune checkpoint") adj2 (inhibit* or block* or therap* or agent*)).ti,ab,kw.

28	ICI*.ti,ab,kw.

29	exp Programmed Cell Death 1 Receptor/

30	programmed cell death protein 1.ti,ab,kw.

31	programmed cell death 1.ti,ab,kw.

32	programmed death 1.ti,ab,kw.

33	PD-1.ti,ab,kw.

34	PD1.ti,ab,kw.

35	programmed death ligand 1.ti,ab,kw.

36	PD-L1.ti,ab,kw.

37	PDL1.ti,ab,kw.

38	cytotoxic t lymphocyte antigen 4.ti,ab,kw.

39	cytotoxic t-lymphocyte-associated antigen 4.ti,ab,kw.

40	cytotoxic t-lymphocyte-associated protein 4.ti,ab,kw.

41	CTLA-4.ti,ab,kw.

42	CTLA4.ti,ab,kw.

43	lymphocyte-activation gene 3.ti,ab,kw.

44	LAG-3.ti,ab,kw.

45	LAG3.ti,ab,kw.

46	pembrolizumab.ti,ab,kw.

47	nivolumab.ti,ab,kw.

48	atezolizumab.ti,ab,kw.

49	durvalumab.ti,ab,kw.

50	avelumab.ti,ab,kw.

51	ipilimumab.ti,ab,kw.

52	tremelimumab.ti,ab,kw.

53	cemiplimab.ti,ab,kw.

54	dostarlimab.ti,ab,kw.

55	relatlimab.ti,ab,kw.

56	toripalimab.ti,ab,kw.

57	tislelizumab.ti,ab,kw.

58	sintilimab.ti,ab,kw.

59	camrelizumab.ti,ab,kw.

60	exp Neoadjuvant Therapy/

61	exp Preoperative Care/

62	neoadjuvant.ti,ab,kw.

63	preoperative.ti,ab,kw.

64	pre-operative.ti,ab,kw.

65	presurg*.ti,ab,kw.

66	pre-surgical.ti,ab,kw.

67	perioperative.ti,ab,kw.

68	"before surgery".ti,ab,kw.

69	"prior to surgery".ti,ab,kw.

70	exp Neoplasms/

71	exp Carcinoma/

72	cancer*.ti,ab,kw.

73	carcinoma*.ti,ab,kw.

74	neoplas*.ti,ab,kw.

75	tumour*.ti,ab,kw.

76	tumor*.ti,ab,kw.

77	malignan*.ti,ab,kw.

78	Or/1-24

79	Or/25-59

80	Or/60-69

81	Or/70-77

82	78 and 79 and 80 and 81

83	limit 82 to (humans)

84	limit 83 to yr="2015 -Current"
