# Supplementary material for: Safety of neoadjuvant radiotherapy or chemoradiotherapy combined with immunotherapy across solid tumors: Systematic review and meta-analysis protocol
Source: PLoS One. 2026 Aug 31;21(8):e0357205. doi: 10.1371/journal.pone.0357205 (PMC13529006; doi:10.1371/journal.pone.0357205)
Supplement: Supplementary file 3 [file pone.0357205.s003.docx]

Dear Editors,

Thank you for considering our new submission, “Safety of neoadjuvant radiotherapy or chemoradiotherapy combined with immunotherapy across solid tumors: a systematic review and meta-analysis protocol.” We appreciate the editorial feedback provided on the earlier version of this manuscript. We found these comments constructive and have substantially revised the protocol to address the concerns raised.

This manuscript is being submitted as a new submission following prior editorial recommendation. In revising the protocol, we focused particularly on strengthening the methodological rigor, transparency, and completeness of the review methods in line with *PLOS ONE* guidance for systematic review protocols. A track changes version of the manuscript has been submitted.

Response to concerns regarding certainty of the body of evidence
We agree that assessment of the certainty of the body of evidence is distinct from study-level risk of bias and is essential for interpretation of review findings. In the revised manuscript, on line 354-370, we have expanded the methods to state explicitly that certainty of evidence will be assessed separately for each critical and important outcome using the GRADE approach. We now clarify that certainty will be judged at the outcome level across the body of evidence and rated as high, moderate, low, or very low based on the GRADE domains. We further specify that the results will be presented in GRADE evidence profile and Summary of Findings tables generated using GRADEpro GDT. This revision was made to ensure that the protocol clearly describes how the certainty of evidence for each outcome will be evaluated and reported.

Response to concerns regarding language restrictions
We also appreciate the concern regarding unjustified language restrictions. In the revised manuscript, we have removed language restrictions from the search strategy and eligibility criteria so that potentially relevant studies will not be excluded on the basis of publication language alone. On line 185 we explicitly note that no language restrictions will be imposed. This change was made to improve the comprehensiveness of the review and to reduce the risk of language bias.

Response to concerns regarding status and timeline of study

As this is a systematic review, participant recruitment is not applicable. At the time of submission, initial study screening and data extraction have been initiated. Data collection is expected to be completed by late July to August 2026, and results are expected by September 2026.

In addition to these specific revisions, we have carefully reviewed the protocol as a whole to improve clarity and reproducibility of the research question, search strategy, eligibility criteria, and planned methods for study selection, risk of bias assessment, and data synthesis.

We believe that these revisions directly address the concerns raised in the previous editorial assessments and substantially strengthen the protocol. We are grateful for the opportunity to submit this revised work for consideration and hope that it will now meet the methodological expectations for publication as a systematic review protocol in *PLOS ONE*.

Sincerely,
Irtaza Tahir MD MSc
On behalf of all authors
